# A Single Transcript Knockdown-Replacement Strategy Employing 5’ UTR Secondary Structures to Precisely Titrate Rescue Protein Translation

**DOI:** 10.3389/fgeed.2022.803375

**Published:** 2022-03-28

**Authors:** Matthew M. Millette, Elizabeth D. Holland, Tanner J. Tenpas, Erik W. Dent

**Affiliations:** ^1^ Department of Neuroscience, School of Medicine and Public Health, University of Wisconsin, Madison, WI, United States; ^2^ Neuroscience Training Program, University of Wisconsin, Madison, WI, United States

**Keywords:** gene therapy, amyotrophic lateral sclerosis, knockdown-rescue, protein translation, hairpin, UTR, RNAi

## Abstract

One overarching goal of gene therapy is the replacement of faulty genes with functional ones. A significant hurdle is presented by the fact that under- or over-expression of a protein may cause disease as readily as coding mutations. There is a clear and present need for pipelines to translate experimentally validated gene therapy strategies to clinical application. To address this we developed a modular, single-transgene expression system for replacing target genes with physiologically expressed variants. In order to accomplish this, we first designed a range of 5’ UTR “attenuator” sequences which predictably diminish translation of the paired gene. These sequences provide wide general utility by allowing control over translation from high expression, ubiquitous promoters. Importantly, we demonstrate that this permits an entirely novel knockdown and rescue application by pairing microRNA-adapted shRNAs alongside their respective replacement gene on a single transcript. A noteworthy candidate for this corrective approach is the degenerative and uniformly fatal motor neuron disease ALS. A strong proportion of non-idiopathic ALS cases are caused by varied mutations to the SOD1 gene, and as clinical trials to treat ALS are being initiated, it is important to consider that loss-of-function mechanisms contribute to its pathology as strongly as any other factor. As a generalized approach to treat monogenic diseases caused by heterogeneous mutations, we demonstrate complete and predictable control over replacement of SOD1 in stable cell lines by varying the strength of attenuators.

## Introduction

Gene knockdown-rescue (KDR) experiments are a mainstay for interrogating protein function. Commonly, production of a wild-type (WT) protein in a cell of interest is silenced *via* RNA interference (RNAi), while simultaneously being replaced by mutated variants. Valuable inferences can be made scrutinizing the cellular consequences of these substitutions. This approach is often used in a preclinical setting to model diseases *in vitro,* especially when patient-derived cell lines are not yet available. For these same diseases an overarching goal of gene therapy is the converse, replacing a mutated protein with a normal protein. We are fortunate in the present era of molecular biology to have many tools at our disposal for manipulating genes and their expression. The discovery of CRISPR/Cas9 and generation of associated tools for genome editing has been a colossal leap forward in this arena (for review, see Hille et al., 2018), though some hurdles do remain in its practical application (Lino et al., 2018; Weisheit et al., 2021). In the ideal circumstance direct editing corrects a mutated sequence, which remains under control of its endogenous promoter. However, there are many situations where this may not be feasible, or where complementary techniques may present significant advantages. For example, challenges are presented to gRNA design by mutations occurring in repetitive regions, within regions conserved across several isoforms, or due to ploidy (Boettcher and McManus 2015). The flexibility of RNAi mechanisms in KDR strategies to target both coding and untranslated portions of a transcript may side-step these obstacles. Additionally, in cases where many different mutations to a single gene cause disease, a generalized KDR approach could benefit a diverse range of patients.

One particularly noteworthy candidate for therapeutic KDR is amyotrophic lateral sclerosis (ALS), a degenerative and uniformly fatal motor neuron disease which, in the US, impacts roughly one person in every 25,000 (Mehta et al., 2014). In 1993 a large-scale, cooperative effort to understand the underpinnings of ALS revealed mutations to Cu/Zn superoxide dismutase (SOD1) as its first known heritable cause (Rosen et al., 1993). While more than 30 genes and loci are now associated with this disease, a strong proportion of cases with known genetic etiology involve SOD1 (Mehta et al., 2014). Remarkably, since this initial discovery more than 180 additional, unique SOD1 mutations have been documented in ALS patients (Zou et al., 2017). SOD1 itself is an enzyme whose canonical role is the protective neutralization of reactive oxygen species within cells. While the preponderance of evidence suggests that abnormal, cytotoxic protein aggregation and mitochondrial dysfunction stemming from SOD1 mutations drives pathology in these cases (Furukawa and Tokuda, 2020; Tak et al., 2020), it is important to note from a clinical standpoint that KO animal studies have shown SOD1 not to be dispensable (Sakellariou et al., 2018). In fact, loss-of-function mechanisms may play as serious a role as any other factor contributing to ALS pathology, so it is therefore unrealistic to expect that simply eliminating mutant SOD1 will be adequate in human gene therapy for ALS [comprehensively reviewed by Kim et al. (2020)]. Thus, it is imperative that we focus instead on the replacement of mutant SOD1 with normal SOD1.

Apart from a vector to introduce such a genetic construct there are two foremost considerations for success: reliable control over rescue gene expression, and effective, stably-tolerated RNAi. Undoubtedly, a significant hurdle is presented by the fact that diseases may be caused as easily by under- or over-expression of a protein as by coding mutations. In addition to disrupting specifically related processes and pathways, ultimately over-expression of any protein should be considered harmful because of the abnormal burden placed on shared cellular resources (Bolognesi and Lehner, 2018). However, as is often the case, we can look to nature for inspiration and solutions. Translational remodeling is a cornerstone of cellular adaptation to changing conditions and is accomplished with fascinatingly varied tools, including RNA-binding proteins, long noncoding RNAs, microRNAs (miRNAs), and post-translational modifications (Gallego and Virshup, 2007; Alquezar et al., 2021; Ho et al., 2021). An active field of research focuses on how sequences encoded in the 5′ UTR of genes contribute to their translational regulation. These range in complexity from simple, thermally-governed steric hindrances to initiation, to permissive or inhibitory switches where cofactor binding allows rapid, spatiotemporally controlled responses (for review, see Leppek et al., 2018). Despite their apparent utility, with the exception of IRES elements, these are rarely incorporated into artificial genetic constructs. In contrast, miRNAs are popularly used on the gene silencing side of the equation. miRNAs are a class of ∼22 nt RNA molecules which cause translational repression or transcript degradation after binding complementary mRNA sequences. Mature miRNAs are processed from precursor stem-loop structures transcribed by RNA Pol II (Fabian et al., 2010; Huntzinger and Izaurralde, 2011). These have been widely adapted into expression cassettes for delivering miRNA-adapted shRNAs, or “shRNAmirs” (Fellmann et al., 2013). This adaptation is highly advantageous in man-made constructs as it permits expression of coding genes and shRNA, encoded in the 3’ untranslated region (UTR), from a single mRNA transcript. A common use is the pairing of fluorescent proteins with shRNAmirs to visually confirm expression of RNAi constructs. Considering the ease of rendering rescue genes insensitive to shRNA, or the ability to target untranslated regions for silencing, it may initially come as a surprise these cassettes are essentially never paired with their respective genes of interest. In practice, this is surely because the ubiquitous promoters used to ensure expression and potency of shRNA in many cell types result in inappropriately high rescue gene expression. It must also be noted that one encounters the opposite problem pairing rescue gene-shRNAmir transcripts with their respective endogenous promoter, as under-expression results from accelerated transcript degradation following shRNA processing (Fellmann et al., 2013).

Fewer than 1 out of 5 clinical trials targeting the central nervous system are successful (Wong et al., 2019). Currently, the number of gene therapy trials pales in comparison to all other clinical trials (∼2,600 vs ∼320,000 as of writing, www.clinicaltrials.gov), but this number has steadily risen year after year. Thus, there is an imminent and incompletely addressed need for robust research tools which are readily translated to clinical application. Toward this goal, we developed a modular, single-transgene expression system for replacing target genes with physiologically matched variants. To accomplish this we generated a standardized library of graded attenuation potency 5’ UTR sequences which predictably attenuate protein translation when driven by a ubiquitous promoter. We characterized the performance of these attenuators with three popularly used promoters: hybrid chicken β-actin (CBh), elongation factor 1-alpha (EF1a), and tet-inducible (Tet-ON). This library of sequences can be used independently to tune translation, but importantly allows pairing of shRNAmir cassettes with their respective rescue gene on the same transcript. This modular design saves valuable packaging capacity in viral vectors and allows functional tethering of rescue expression and silencing with inducible promoters. As proof of concept as a gene therapy construct for ALS, we demonstrate complete and reliable control over rescue SOD1 protein translation in stable cell lines by varying the strength of attenuators. We present this complete “pKDR” toolkit for use, anticipating it will accelerate basic research, and since they are readily transferable between vectors, also establish a pipeline for translating validated corrective strategies to therapeutic vectors.

## Materials and Methods

### Hairpin Attenuator Sequences

A multi-step process was required to generate attenuator sequences. We began with a foundation of seven of eight nucleotides (GCGGCCG) from a NotI digest site, serving as a highly thermally stable base and sharing six nucleotides with a Kozak consensus sequence. This facilitated cloning and permitted the placement of the hairpin as close to the start codon as possible without design compromises. An internal, non-complementary loop with the sequence TATACT is shared across all structures. Using the EGNAS program (Kick et al., 2012), we then generated a comprehensive list of high thermal stability stem sequences to append (see EGNAS config. txt and forbidden. txt in Supplementary Materials). We limited sequences to 11 nt in length, with one single A/T pair, and no self-complementarity. These parameters were chosen after manual optimization, and considered work by Babendure et al., 2006 which illustrated a precipitous drop in translation between −20 and −40 kcal/mol caused by high G/C content hairpins. In total, this resulted in 1,360 unique sequences, from which lower stability attenuator structures were created by removing one innermost nucleotide pair at a time. After removing duplicate sequences and those with 100% G/C stems, the remaining 5,729 were modeled to assess initial free energy (initial dG, kcal/mol) using mFold default parameters (Zuker, 2003). This range covered −15.7 to −48.2 kcal/mol. Finally, based on our preliminary results shown in Supplementary Figure S1, we estimated a range of −27 to −40 kcal/mol would yield attenuators from roughly 50–10% original promoter strength, and manually selected a subset spanning this range in uniform increments averaging 0.54 kcal/mol for experimental evaluation. The complete list of structures is available in the following archive: https://bit.ly/millette_frontiers_2022_hairpin_generation.

### pKDR Plasmid Design and Construction

pKDR plasmids were assembled using standard molecular cloning techniques including PCR amplification, restriction digest, and Gibson assembly. Two primary versions of the KDR construct were created. From 5′ to 3’, the larger construct consists of a hairpin attenuator sequence, Kozak consensus sequence, rescue gene, IRES element, mScarlet (Bindels, et al., 2017) membrane affinity label (N-terminal GNAI2 fragment, Clift et al., 2017), and miRE cassette (Fellmann et al., 2013). A “minimal” version was also created without the IRES or fluorescent indicator. The backbone used for the Tet-On is identical except for the inclusion of a separate hPGK promoter and constitutively produced rTTA gene. Attenuator sequences were ordered from IDT as single-stranded ultramers with overhangs complementary to the destination vector. These were inserted into AgeI and NotI linearized backbone using NEB Hifi master mix according to the manufacturer provided protocol. Rescue genes were inserted using NotI and SbfI sites. shRNAs were designed using the splashRNA utility (Pelossof et al., 2017), ordered from IDT as ultramers with complementary overhangs and inserted *via* Gibson assembly into the miRE cassette digested with EcoRI and XhoI. Complete maps may be found on Addgene for each respective plasmid, or are available by reasonable request. Key DNA sequences and their sources are available in Supplementary Table S2.

### HEK293T Cell Culture and Transfection

HEK293T cells were maintained at 37°C, 5% CO_2_ in standard HEK media comprised of DMEM High Glucose (Gibco), sodium pyruvate (Gibco), 10% FBS, and supplemented with 1% Primocin (Invivogen). Cells were passaged at ∼80% confluency and were used both for experiments and lentivirus production. All transfections used Transporter 5 (Polysciences) PEI reagent following the manufacturer’s protocol. Cells expressing Tet-ON promoter constructs were maintained in media containing 1 ug/uL doxycycline (Sigma-Aldrich).

### Imaging and Analysis

Initial quantification of both attenuator performance and shRNA efficacy was carried out by fluorescence ratiometry. For attenuators, the KDR constructs themselves were used for analysis by cloning a GFP (mClover3) gene into the “rescue” position. The impact of each attenuator on this GFP fluorescence was ratioed against independent, IRES-mediated RFP (mScarlet-i) expression. HEK293T cells were plated in 24-well chambers at a density of 0.05 × 10^6^ per well. Cells were cultured for 48 h before transfection with 250 ng plasmid DNA. Cells were fixed 24 h later using 4% paraformaldehyde-KREB-sucrose (PKS) maintained at 37 °C (Dent and Meiri, 1992). Images were captured using a Zeiss LSM800 using an EC Plan-Neofluar 10x/0.30 M27 objective and 488 and 568 nm excitation lasers. 4 × 4 tiled images were taken at 16 bit depth at software-determined optimal resolution, yielding a final composite size of 948 × 948 pixels/2366 × 2366 microns. To analyze these image sets we wrote software in Python which executes pixel-wise comparisons of GFP to RFP intensity provided under MIT License at https://github.com/MattMillette/Pixelwise-Fluorescence-Ratiometry. CZI files were converted to lossless TIFFs with FIJI (Schindelin et al., 2012) then, to speed processing, composites were subdivided into 100 equivalently sized images using ImageMagick utility (imagemagick.org). Replicate averages reported are each greater than 1,000 transfected cells. Background noise thresholds were manually determined prior to analysis and applied uniformly to all image sets.

### Lentivirus Production, Cell Line Generation

All steps were performed under sterile BSL-2 conditions. Briefly, HEK293T packaging cells were seeded at a density of 3.8 × 10^6^ cells per plate in complete DMEM in 10 cm tissue culture plates. After ∼20 h incubation media was aspirated and replaced with Opti-MEM (Gibco). Cells were then cotransfected with psPAX2 (1.3 pmol), pMD2. G (0.72 pmol), and pKDR-SOD1 transfer plasmid (1.64 pmol). Following overnight incubation media was again aspirated and replaced with standard HEK media. 48 h later virus-containing media was collected into 15 cm conical tubes (BD Falcon) and centrifuged at 500 rcf for 10 min. Viral supernatant was filtered through a 0.45 μm PES filter (Corning), snap frozen in liquid nitrogen and stored at -80°C. For cell line generation, HEK293T cells were seeded at a density of 0.3 × 10^6^ per 6-well chamber and incubated ∼24 h before complete exchange with 2 ml of virus-containing media. The following morning media was again exchanged, and 48 h elapsed before beginning puromycin (Sigma-Aldrich) selection protocol. Kill curve experiments determined 5ug/mL concentration eliminated all untransduced cells after 3–4 days. Remaining cells were then maintained in puromycin-containing media until reaching confluency, then passaged normally thereafter. Transgene copy number was estimated for the unattenuated control line using ddPCR and is included as a supplemental report (see Supplementary Material).

### Western Blot Analysis

SOD1 KO and KDR cell lines were generated as described above. Cells were plated at a density of 0.3 × 10^6^ in 6-well plates and grown to confluency. Cultures were washed once with cold PBS, then lysed with 300 µl NP-40 Lysis Buffer (Invitrogen) with Complete Mini (Roche). Lysate was spun at 21,000 g for 10 min at 4°C and supernatants were flash-frozen and stored at -80°C until use. Samples were thawed and loaded into a 4–15% SDS-Page gel (Bio-Rad), then transferred to PVDF membrane (Millipore). Membranes were blocked in 5% milk in 0.1% TBS-T, incubated with primary antibody overnight at 4°C, and blotted with a horseradish peroxidase (HRP) conjugated secondary antibody at room temperature for 1 h. Antibodies used were rabbit anti-Superoxide dismutase 1 (1:2,500, ab13498, Abcam), recombinant rabbit anti-beta IV Tubulin (1:5,000, ab179509, Abcam), and goat anti-rabbit HRP (1:5,000, 31,460, Thermo Scientific). Protein bands were visualized using Pierce ECL Western blotting substrate (Thermo Scientific) and quantified using FIJI following documentation for densitometry (https://imagej.nih.gov/ij/docs/menus/analyze.html#gels). Entire blots are shown in Supplementary Figure S3.

### Graphing and Statistics

Graphpad Prism was used for all graphing and statistical analyses. Analysis was conducted on a minimum of three biological replicates.

## Results

### Establishment of a Graded-Strength Library of Translation Attenuator Sequences for a Novel Gene Expression and Replacement Platform

**FIGURE 1:**
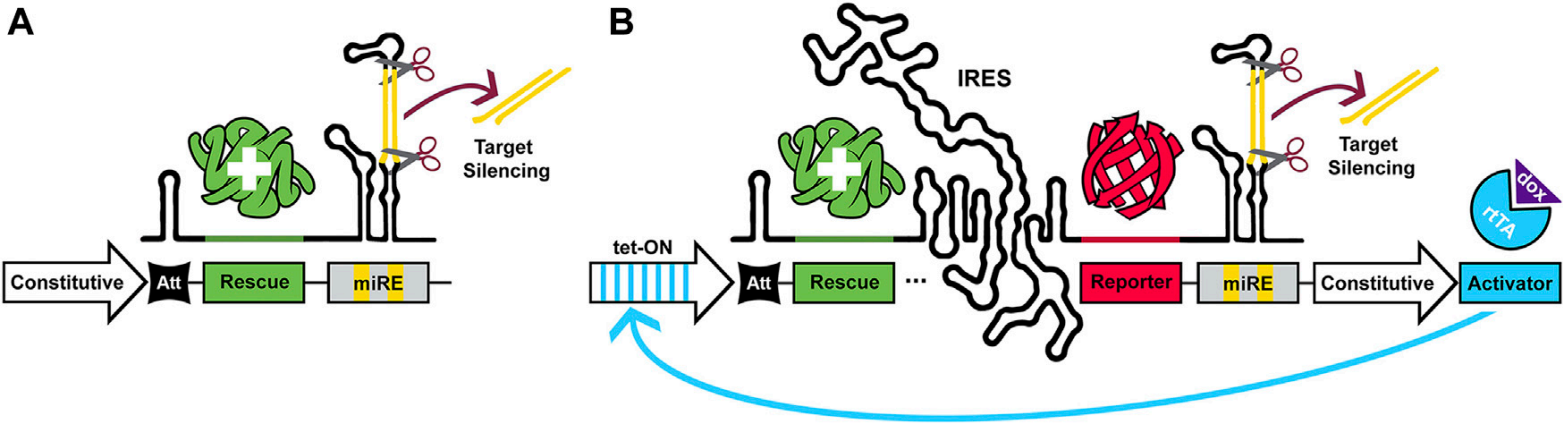
Single-transgene constructs for gene silencing and physiologically matched replacement. Schematics illustrating two possible configurations of pKDR constructs. **(A)** Represents the minimal, constitutive construct without fluorescent reporter, which consists of a constitutive promoter, an attenuator (Att), a rescue sequence and a miRNA-adapted shRNA (miRE). **(B)** Represents the inducible variant with IRES and reporter inserted between the rescue sequence and the miRE sequence. A single-transgene approach to gene replacement delivers shRNA to silence endogenous expression, while allowing the system to be driven interchangeably by either constitutive and/or inducible promoters and offering control over translation level with choice of attenuator (Att) strength.

MRNA stem-loop structures, or hairpins, are one of many distinct types of regulatory elements which may be encoded in the 5′ UTR of genes (Leppek et al., 2018). It has been demonstrated that, upon encountering the ribosome, hairpins attenuate translation through steric hindrance of tRNA binding and inhibiting translation elongation (Bao et al., 2020). Ribosome stalling may also contribute to attenuation *via* mRNA decay (Xie and Chen, 2017). These structures stochastically alternate between open/permissive and closed/inhibitory conformations (Bercy and Bockelmann, 2015). The potency of this attenuation is predominantly governed by an inverse relationship with thermal stability, but other influential properties include their position relative to both the 5′ cap and initiation codon (López de Quinto and Martínez-Salas, 1998; Babendure et al., 2006, and reviewed in; Xie and Chen, 2017). At the outset of this project we wished to devise a system which, for a chosen gene, would use these hairpins to physiologically match its endogenous expression by attenuating high copy, ubiquitous promoters. Further inclusion of a 3’ UTR microRNA-adapted shRNA expression cassette would then allow silencing of the chosen gene, resulting in knockdown and replacement from a single mRNA transcript. This saves on limited viral packaging capacity, and has the added benefit of being inducible and reversible if driven by a Tet-ON, or functionally similar promoter. Examples of these constructs with and without fluorescent reporters are schematized in Figure 1.

**FIGURE 2:**
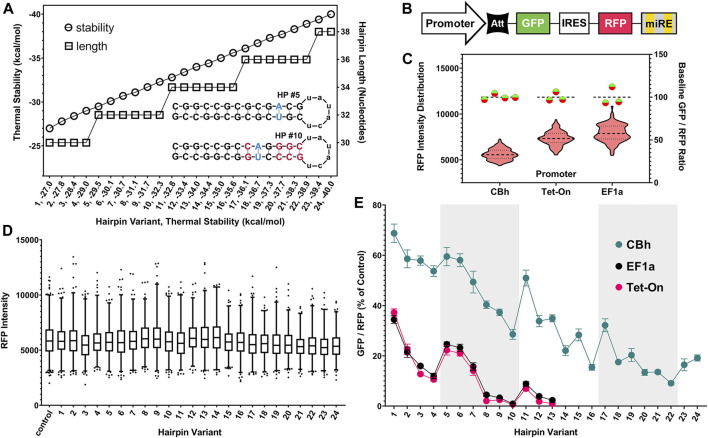
Generation of hairpin structure library and assessment of performance as translational attenuators using fluorescence ratiometry. **(A)** Hairpin (HP) “attenuator” sequence stability gradient pictured (circles, left axis) with corresponding length (squares, right axis). Thermal stability is the primary determinant of attenuator function and is inversely correlated with protein translation from paired genes. Inset structure diagrams highlight sequence shuffling used to produce attenuators of differing stability, but with identical length and GC content. The final subset tested was selected from a comprehensive list of >5,700 unique sequences, which maximized uniformity of coverage within the functionally relevant range. **(B)** Simplified schematic of knockdown-replacement plasmid (pKDR) construct, displayed here with a GFP (mClover3) gene in place of the “rescue” gene. Validation experiments to ratiometrically assess efficacy of hairpins as translational attenuators were carried out with non-targeting luciferase shRNA cloned into the 3′ endogenous mir30 (miRE) cassette. **(C)** Baseline performance of CBh, EF1a, and tetracycline-inducible (Tet-On) promoters after transient plasmid transfection in HEK293T cells. Violin plot displays RFP (mScarlet) intensity distribution across all images taken for each promoter (left *Y* axis). Baseline un-attenuated GFP/RFP intensity ratios are plotted on the right *Y* axis (Green/Red circles). While base expression strength differs between promoters, each un-attenuated control construct provides a consistent internal baseline measurement. **(D)** IRES-mediated translation is unimpacted by 5′ hairpins, as RFP intensity is uniform across the entire range of attenuator sequences after transient plasmid transfection in HEK293T cells. **(E)** Increasing hairpin stability attenuates GFP translation in non-linear, but highly internally consistent and reproducible manner. Alternating white and gray regions on the graph indicate changes in lengths of hairpin (30, 32, 34, 36, and 38 from left to right). Ratios for each promoter are displayed as the percent of their own un-attenuated control. While the CBh promoter is resistant to attenuation, all promoters mirrored a nonlinear performance trend after transient plasmid transfection in HEK293T cells.

Preliminary testing in B35 rat neuronal cells quickly determined that stepwise removal of nucleotide pairs from the hairpin sequence would not yield the uniformity or gradation of attenuation required for a robust system (Supplementary Figure S1). We determined that the most effective way to achieve this was a comprehensive modeling approach. Figure 2A graphically summarizes the approach taken and its benefits. We began by using the EGNAS program to create an exhaustive list, then repeated the “trimming” procedure to yield an exhaustive coverage of all possible sequences given pre-set constraints. We allowed one single A-T pair (yielding an A-U pair in the mRNA stem) to maximize uniformity of G-C content. This yielded 5,729 unique structures with stability between −15 and −48 kcal/mol, which we had identified in preliminary *in vitro* testing to be a functionally relevant range (Supplementary Figure S1). We selected 24 sequences from this master list, with an effort to maximize linearity of the stability gradient. Circles in Figure 2A illustrate the linearity of stability, relative to the length of the sequence itself, shown with squares. Two examples of hairpin structures (HP #5, HP #10) are shown under the thermal stability and hairpin length lines in Figure 2A. Note that both hairpin structures have the same number of nucleotides 32) and identical GC content, but very different thermal stabilities (−29.5 kcal/mol vs. −32.3 kcal/mol). Relaxing constraints by allowing, for example, more than one A-T pair would increase the number of resulting sequences in an exponential manner. In principle, hundreds of thousands of different sequences could fall within this useful range.

**FIGURE 3:**
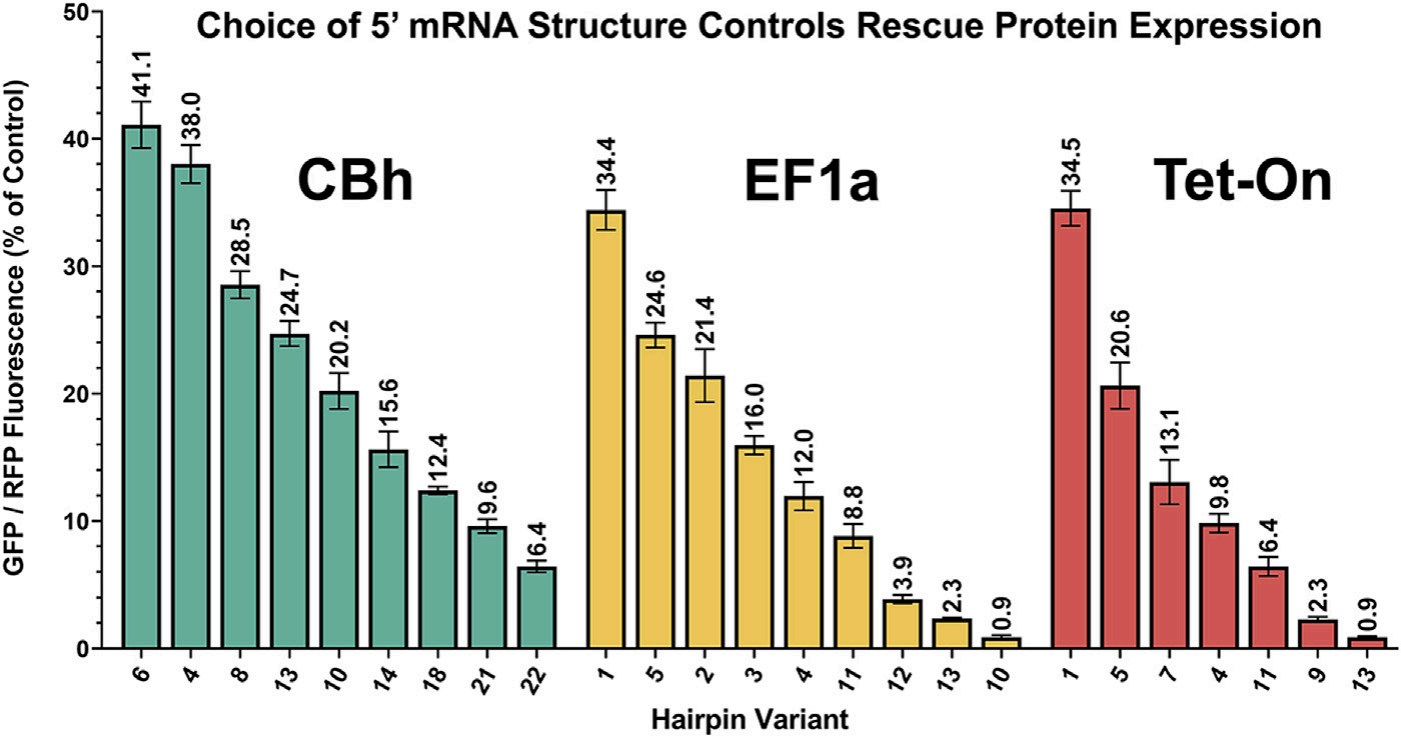
Performance-ranked collection of select hairpins. Scalable translation in Knockdown-Replacement vectors allows endogenous expression matching when employed with constructs in Figure 1. Pictured are a core subset of constructs from Figure 2E, ranked by performance rather than thermal stability, and normalized to the brightest average RFP signal from the EF1a promoter. Modular selection of hairpin structure controls protein translation, permitting physiological matching of “rescue” gene expression to endogenous levels. All of these plasmids (9 CBh, 9 EF1a, 7 Tet-On) are deposited with Addgene.

A simplified schematic of the ratiometric plasmid used to measure attenuator performance is shown in Figure 2B. This genetic construct is identical to that used for KDR except here GFP is substituted in the “rescue” gene position and non-targeting control shRNA is expressed. Baseline performance of this setup with no attenuator is shown for CBh, EF1a and Tet-On promoters after transient plasmid transfection in HEK293T cells (Figure 2C). HEK293T cells were used throughout the rest of the manuscript because their flatter and broader morphology in culture was more amenable to our imaging strategy than B35 cells. Red violin plots show RFP intensity for all cells quantified and green/red circles show virtually identical GFP/RFP expression ratios regardless of promoter. Moreover, since the RFP is produced from an IRES site it is unimpacted by the attenuator, and thus serves as an ideal internal control (Figure 2D). When expressing the full range of these constructs *via* transient plasmid transfection in HEK293T cells we found a curiously nonlinear inverse relationship between thermal stability and translation of GFP (Figure 2E). Interestingly, we identified a clear trend within subsets of hairpin length, where attenuation “relaxes” each time hairpin length is increased, despite steadily increasing thermal stability. We have highlighted these subsets using alternating gray and white bars to illustrate where changes in length occur. Importantly, though nonlinearity suggests additional relationships may yet be described, each individual hairpin was highly consistent across replicates. This consistency allows us to rank attenuators by performance for practical use. Out of the entire library of 24 constructs we chose seven to nine that resulted in relatively linear performance in attenuating protein expression (Figure 3), where CBh and Tet-ON performance are scaled to the highest expressing EF1a for ease of functional comparison.

### Replacement of SOD1 Expression is Effectively and Predictably Controlled by Choice of Attenuator

**FIGURE 4:**
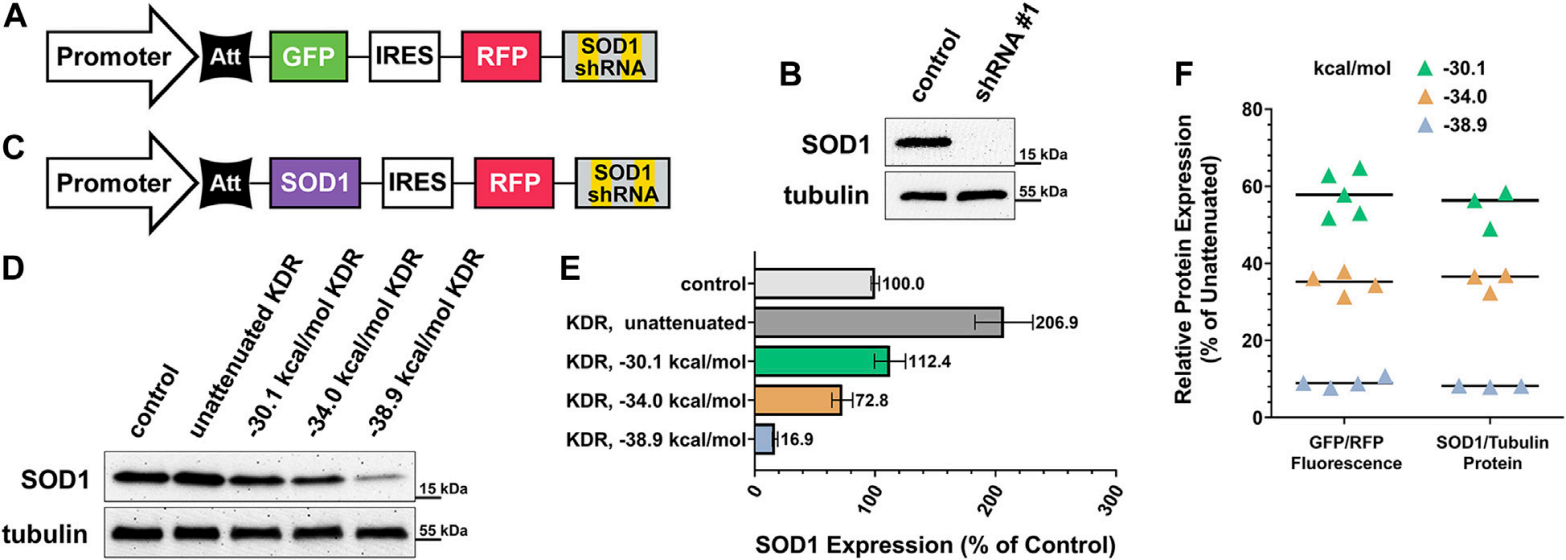
Practical application of knockdown-replacement strategy using pKDR-SOD1 virus. **(A)** SOD1 gene specific shRNA potency was validated using lentivirus carrying SOD1 shRNA, but GFP coding sequence in rescue position rather than SOD1. **(B)** Cell lines were created using SOD1-KD virus as previously described, then evaluated by western blot. Comparison of KD condition (equivalent to background noise) to un-transduced control HEKs confirms complete shRNA efficacy. **(C)** GFP gene was then exchanged for SOD1 and a range of graded-expression constructs were created, this included un-attenuated rescue expression (no hairpin) and three levels of increasing attenuator potency (−30.1, −34.0, and −38.9 kcal/mol). **(D)** Cell lines were created for each of these 4 conditions, then SOD1 expression was evaluated by western blot and quantified in **(E)**. Un-attenuated SOD1 expression was found to be over twice as high as baseline expression, whereas the −30.1 kcal/mol construct most closely matched endogenous expression. **(F)** Plasmid-based ratio of GFP “rescue”/RFP internal control fluorescence to virally expressed SOD1 rescue/endogenous tubulin protein is functionally identical for each attenuator tested. At −30.1 kcal/mol mean GFP expression was 58.05% of control, SEM ±2.58, 95% CI ± 5.05%, and SOD1 mean = 54.56%, SEM ±2.85. At −34 kcal/mol mean GFP expression was 34.91%, SEM = 1.40, 95% CI ± 2.74%, and SOD1 mean = 35.27%, SEM ±1.46. At −38.9 kcal/mol mean GFP expression was 9.10%, SEM ±0.64, 95% CI ± 1.25%, and SOD1 mean = 8.14%, SEM ±0.11. Means for each SOD1 attenuation condition fall within the 95% confidence interval of their corresponding plasmid-based *in vitro* measurement.

Over 180 unique, causative mutations to SOD1 have been identified in ALS patients (Zou et al., 2017). Considering this impressive heterogeneity, a one-size-fits-all approach to gene therapy for these individuals would be of enormous benefit. We sought a proof of principle demonstration that any mutant variant of this gene could be knocked down and replaced with normal SOD1 using our KDR approach. Figure 4A illustrates the construct used to validate our choice of shRNA to silence the SOD1 gene. This construct, containing a CBh promoter, is identical to that used throughout Figure 2 with the exception of SOD1-targeted shRNA, rather than control shRNA, and that it was cloned into a lentiviral transfer vector. We also designed a complementary ratiometric construct for use in testing the knockdown efficiency of shRNA sequences. SOD1 shRNA candidates were selected using the SplashRNA web utility (Pelossof et al., 2017) and were pre-evaluated *in vitro* using this shRNA ratio plasmid (Supplementary Figure S2A). Two candidate shRNAs performed equally as well as three published positive control shRNAs to other genes (Supplementary Figure S2B). A HEK293T cell line was generated using the knockdown-only lentivirus (shRNA #1, targeting the 3’ UTR of SOD1) schematized in Figure 4A, and then shown by western blot to completely eliminate endogenous SOD1 expression (Figure 4B).

Having confirmed shRNA efficacy we then replaced the GFP placeholder gene with the coding region of WT SOD1, schematized in Figure 4C, to evaluate attenuation performance against a SOD1-depleted background. The primary goal of this experiment was to confirm that plasmid-based measurements of attenuation and expression (Figure 3) are predictive when the KDR construct is genomically integrated *via* lentiviral infection. To accomplish genomic integration we inserted three hairpins of uniformly increasing stability into three lentiviral SOD1 KDR constructs ((#6) −30.1 (#13) −34.0, and (#22) −38.9 kcal/mol) and created three additional cell lines containing each construct. These cell lines were puromycin-selected then re-expanded. Thus, expression levels reported are of heterogeneous population averages. Figure 4D shows the western blot assessment of the resulting SOD1 expression range alongside control and un-attenuated conditions. This blot is quantified in Figure 4E, showing a uniform decrease in SOD1 replacement expression with increasing attenuator strength. In Figure 4F comparisons are made to our earlier, plasmid-based assessment (Figure 2E) of the same attenuators to real world SOD1-KDR performance of genomically integrated constructs. Critically, we found that initial GFP/RFP fluorescence-based readouts of expression were functionally identical to SOD1/Tubulin protein ratios. These data affirm that the library of constructs provided (Figure 2E, with a ranked subset in Figure 3) can be used in a routine and reliable manner to tune expression for other proteins, in other applications.

## Discussion

Here we present a novel knockdown-rescue strategy for substituting target protein expression with physiologically matched replacements. The utility of this system is multi-fold, potentially spanning the complete research process from preliminary experimentation to clinical application. An ongoing and unmet need for such tools to manipulate protein translation has been highlighted by prior, alternate strategies (Morita et al., 2012; Koh et al., 2013). A key aspect of our approach was our construction and implementation of a graded-strength library of hairpin sequences capable of titrating protein expression. Observations that mRNA hairpins in the 5’ UTR influence translation predate the current millennium (López de Quinto and Martínez-Salas, 1998), however, with the present work we are the first to put this knowledge to practical application. If others previously recognized the same potential utility, we suspect the obstacles we encountered may have frustrated earlier efforts. Chiefly, we discovered that the functionally useful range of attenuation occupies a narrow margin of thermal stability, spanning as few as six nucleotide pairs in length. Additionally, despite designing sequences to be as uniform in composition as possible, there exists an imperfect association between modeled stability and actual attenuation, which is further exacerbated at this minute level. In total, this made the generation of a linear gradient particularly challenging. We overcame these obstacles by employing a comprehensive modeling approach, high throughput *in vitro* assessment of reporter gene expression, and ultimately generation of a performance-ranked library of attenuators for both constitutive and inducible promoters. The result is a powerful and flexible system for gene manipulation.

As proof of principle treatment for monogenic disorders we have demonstrated complete silencing and adjustable, predictable replacement of SOD1 in stable cell lines. We selected SOD1 because a strong proportion of non-idiopathic Amyotrophic Lateral Sclerosis (ALS) cases are caused by widely varied mutations to the SOD1 gene (Zou et al., 2017). ALS itself is a debilitating neurodegenerative disease with few treatments and no cure. As gene therapy trials for ALS are being initiated, notably including siRNA-only strategies, it seems prudent to consider that loss-of-function mechanisms may contribute to ALS pathology as strongly as any other factor (Kim et al., 2020). Since SOD1 cannot be assumed to be dispensable in humans, and animal studies suggest it is not (Sakellariou et al., 2018), our construct may be ideal for replacing any mutated variant with appropriate levels of normal SOD1 protein. Indeed, this one-size-fits-all approach may provide particularly great utility in treating other diseases with similarly heterogenous genetic origins as ALS. Critically, genomically integrated performance of individual attenuator sequences is internally consistent with initial plasmid-based estimations, which permits this system to be intelligently adapted to other diseases of known genetic origin. Taken together, these experiments serve to confirm that our library of constructs behaves as anticipated in actual application. Naturally, for each new cell type, target protein, or promoter a similar process of optimization will be necessary. Though this current work primarily highlights the potential utility of this approach in gene therapy, we anticipate our standardized library for fine-tuning gene expression may see application in cell engineering and synthetic biology as well. We expect future implementations may take advantage of safe harbor loci (using CRISPR-Cas based tools) for additional measure of control and safety. It will be exciting to see whether future iterations can be expanded *via* computational biology or machine-assisted design.

## Data Availability

The original contributions presented in the study are included in the article/Supplementary Material, further inquiries can be directed to the corresponding author.
