# Supplementary material for: A Single Transcript Knockdown-Replacement Strategy Employing 5’ UTR Secondary Structures to Precisely Titrate Rescue Protein Translation
Source: Front Genome Ed. 2022 Mar 28;4:803375. doi: 10.3389/fgeed.2022.803375 (PMC8995503; doi:10.3389/fgeed.2022.803375)
Supplement: Supplementary file 1 [file DataSheet2.pdf]

Droplet Digital PCR
Droplets generated on a Bio-Rad Manual Droplet Generator.
20 uL of template, primers and EvaGreen supermix and 70 uL of EvaGreen droplet generation oil added to cartridge sample and oil wells respectively.
Makeup of samples:

Solution	Concentration	Volume added	Final Concentration
Template DNA	1:100 or 1:10	1 uL	1:2000 or 1:200
Forward Primer	2 uM	1 uL	100 nM
Reverse Primer	2 uM	1 uL	100 nM
EvaGreen Supermix	2X	10 uL	1X
Water	-	7 uL	-

Droplets were transferred to a 96-well PCR plate, heat sealed with foil and added to a Bio-Rad C1000 Touch thermal cycler.
PCR cycling protocol:

Cycling Step	Temperature	Time	Ramp Rate	# Cycles
Enzyme activation	95 C	5 min		1
Denaturation	95 C	30 sec		40
Annealing/extension	60 C	1 min	~2C/sec	
Signal stabilization	4 C	5 min		1
	90 C	5 min		1
Hold	4 C	Infinite		1

Droplets were subsequently read on a Bio-Rad QX200 droplet reader. Samples had between 9000-18000 droplets and exhibited good differentiation between positive and negative droplets.

We used the RPPH1 gene as a well-established single copy reference against which to compare the puromycin resistance gene found in our viral construct. *(See PMIDs: 26874951, 2308839, 31164119).* Template DNA from unattenuated control cell line. Primers used are listed below, and following data illustrate copies of puromycin are found in relative equivalence to single-copy RPPH1 gene.

1 GCAACCTCCCCTTCTACGAGC Puro C-terminal Forward
2 GGTCTTAAAGGTACCTCAGGC Puro C-terminal Reverse1
3 GGCTAAGATCTACAGCTGCC Puro C-terminal Reverse2
4 CGCGCGAGGTCAGACT RPPH1 Reference Gene Forward
5 GGTACCTCACCTCAGCCATT RPPH1 Reference Gene Reverse

		Using 1 uL 1:100 dilution	*Using 1uL 1:10 dilution*	*From 1:100 dilution*	*From 1:10 dilution*
**Primer Set**	**Description**	**# of copies/ 20uL well**	**# of copies/ 20uL well**	**# of copies/ uL of stock**	**# of copies/ uL of stock**
4+5	Reference	14.3	162	1430	1620
1+2	Puromycin	11.8	151	1180	1510
1+3	Puromycin	13	145	1300	1450

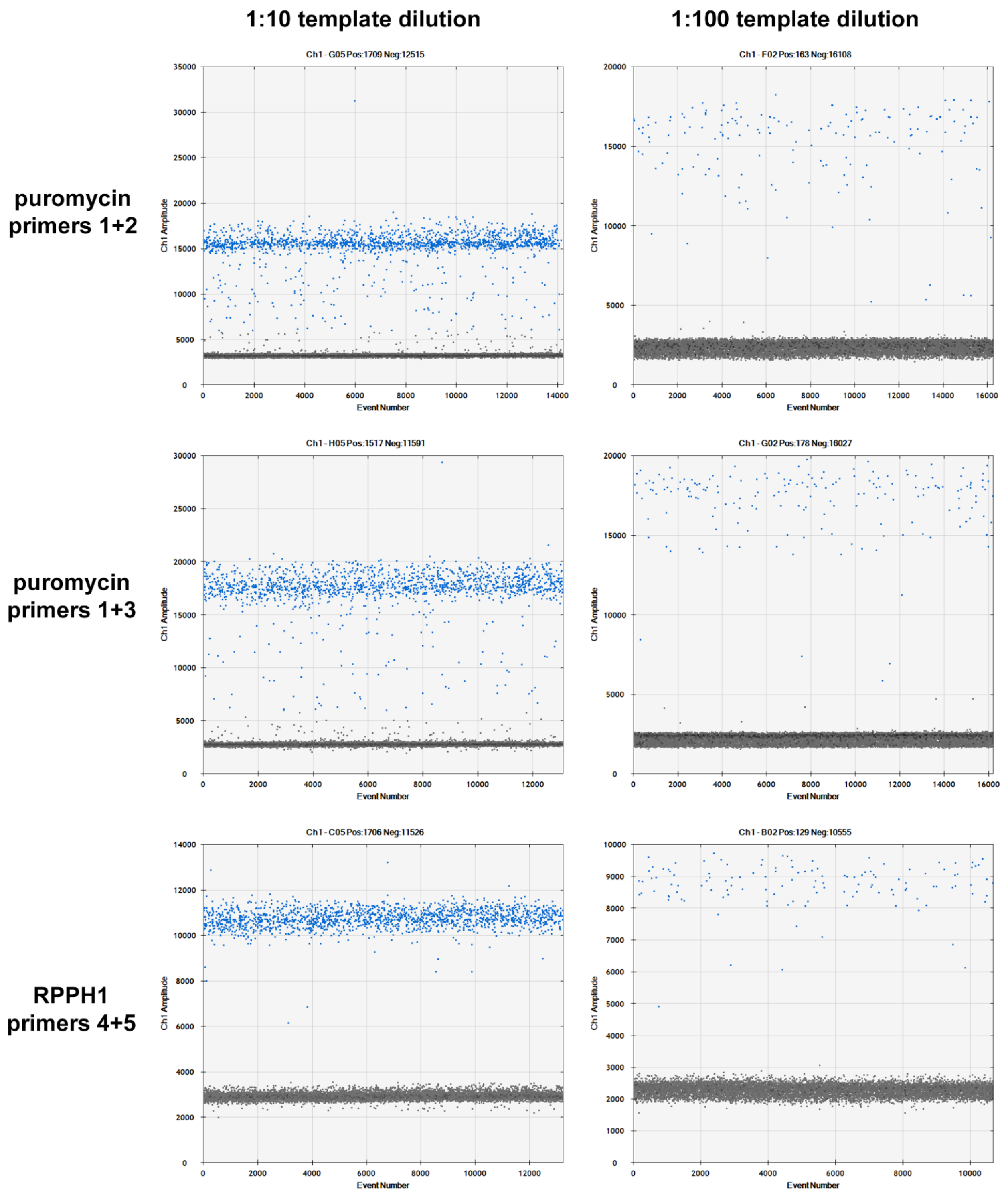
1:10 template dilution
1:100 template dilution
puromycin primers 1+2
puromycin primers 1+3
RPPH1 primers 4+5
Ch1 - G05 Pos:1709 Neg:12515
Ch1 - F02 Pos:163 Neg:16108
Ch1 - H05 Pos:1517 Neg:11591
Ch1 - G02 Pos:178 Neg:16027
Ch1 - C05 Pos:1706 Neg:11526
Ch1 - B02 Pos:129 Neg:10555
Ch1 Amplitude
Event Number
