# Supplementary material for: A Single Transcript Knockdown-Replacement Strategy Employing 5’ UTR Secondary Structures to Precisely Titrate Rescue Protein Translation
Source: Front Genome Ed. 2022 Mar 28;4:803375. doi: 10.3389/fgeed.2022.803375 (PMC8995503; doi:10.3389/fgeed.2022.803375)
Supplement: Supplementary file 2 [file Table1.docx]

Supplementary Materials and Methods


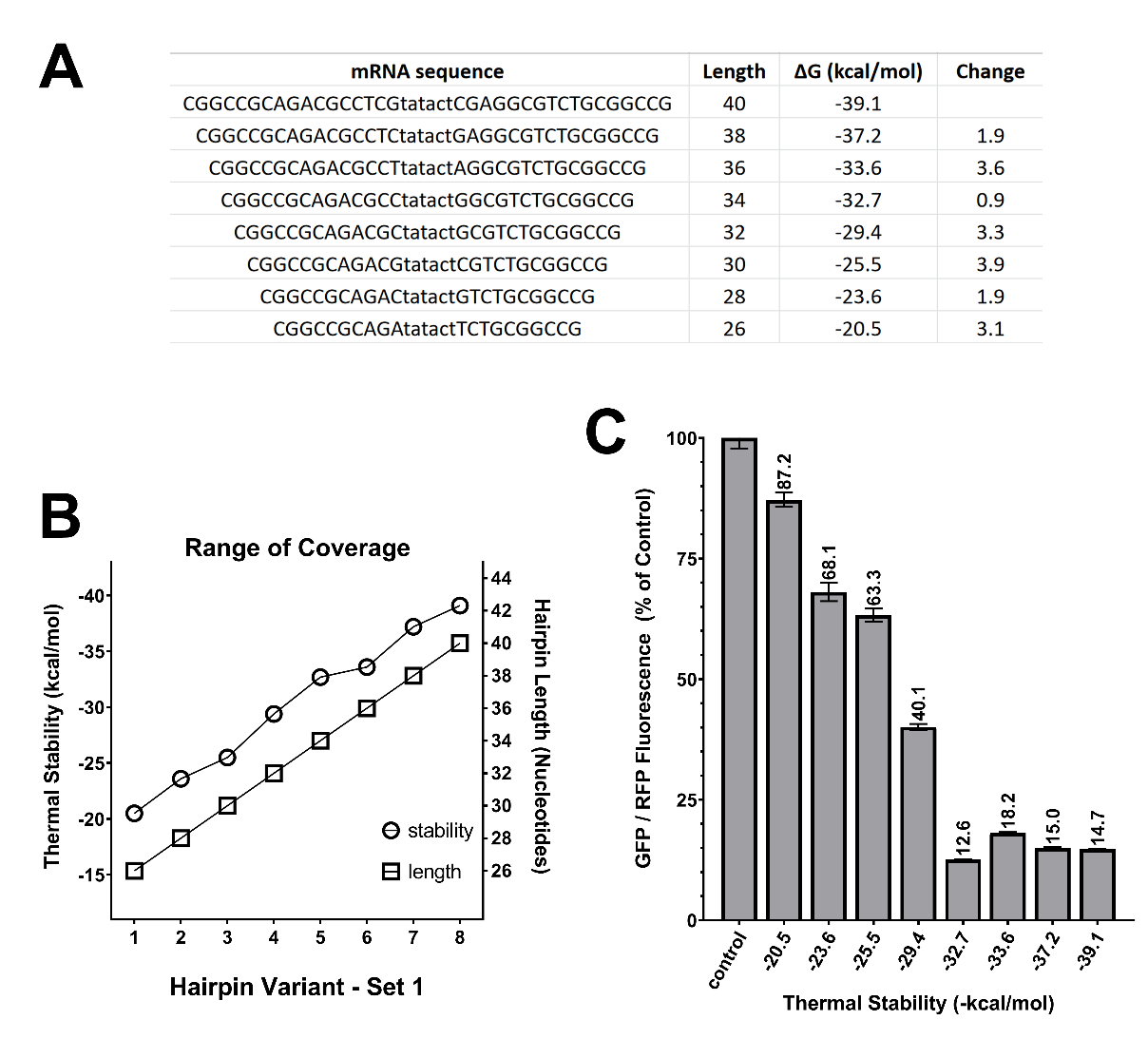


**Supplementary Fig 1) Preliminary Attenuation Curve Measurements**

The following process was used for initial estimation of practically useful thermal stability range. Referencing work done by Babendure et al. (2006) which identified a precipituous drop in translation between -20 and -40 kcal/mol, we first manually-generated a single hairpin sequence of 40nt length and thermal stability of -39.1 kcal/mol. We shortened this sequence one nucleotide pair at a time to create a gradient of thermal stability, stopping at the desired lower bound of -20.5 kcal/mol (A). Thermal stability versus length is plotted in (B). We then used the same ratiometric plasmid pictured in Fig. 1B to evaluate the impact of this range of translational attenuators. Only the CBh promoter was used for this analysis. Briefly, B35 rat neuronal cells were cultured and transfected with ratiometric plasmids carrying the range of attenuators shown in (A) in the same manner as described for HEK cells in the primary methods. Immediately before analysis, cells were released from culture plates using Trypsin-EDTA 0.05% (Gibco), quenched with 10% serum-containing culture medium, centrifuged at 400 rcf for 5 minutes, resuspended to a final cell concentration 1x10^7^ cells/mL, then strained to achieve a single cell suspension. GFP and RFP intensities for each cell were measured using a ThermoFisher Attune flow cytometer, and data was processed using FlowJo Single Cell Data Analysis Software. DAPI used as exclusion marker for non-viable cells. Average ratios shown in (C) are representative of, at fewest, 14837 viable cells per condition. Based on these data, we estimated that a range from -27 to -40 kcal/mol would provide us with attenuators ranging from roughly 50% to 10% original promoter strength.


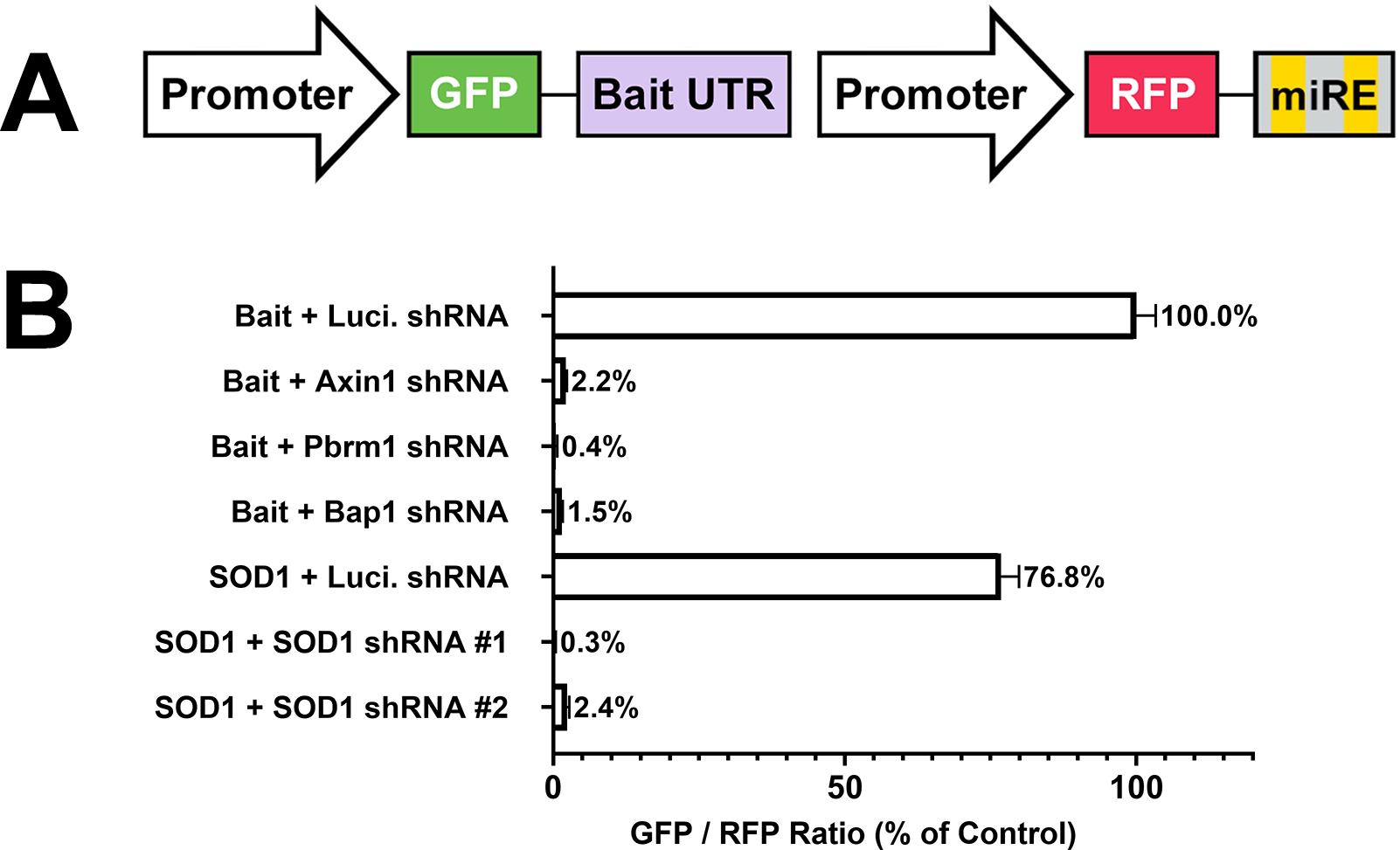


**Supplementary Fig 2) shRNA Ratio Plasmid Design and Performance**

(A) This is a dual promoter plasmid containing a GFP fusion protein of a partial, full or 3’ UTR sequence for a gene of interest. In the same plasmid RFP is produced with a miRNA sequence directed at the sequence fused to GFP. (B) By ratioing the levels of GFP fusion protein to RFP the potency of the shRNA knockdown can be discerned.

For estimation of shRNA potency we built an additional internally controlled, ratiometric plasmid. In contrast to single transcript KDR constructs, in this case a dual CBh promoter setup produced separate GFP-bait and RFP-shRNA transcripts. This is necessary to keep red “housekeeper” intensity functionally independent from shRNA efficacy. To establish best case readout of GFP silencing three positive control targets were selected (Axin1, Pbrm1 and Bap1), each of which were previously demonstrated to have 99%-100% efficacy by western blot analysis (Pelossof et al., 2017). An artificial “Bait” 3’ UTR attached to GFP was constructed from 22 bp fragments of Bap1, Pbrm1, and Axin1 genes. *Ren. Luciferase* non-targeting shRNA was paired with this same bait as a negative control. SOD1 efficacy was estimated by instead attaching the entire open reading frame of SOD1 as a 3’ UTR of GFP. In a manner identical to the positive controls, the SOD1 ORF was then targeted by SOD1-specific shRNA cleaved from the second, separate RFP-coding transcript. A complete list of sequences is available in supplementary table 2. In Fig. 4B we validate performance by showing agreement between the GFP/RFP ratio of a GFP-SOD1 fusion protein and levels of SOD1 through western blotting. However, it is worth noting this plasmid is especially useful for knockdown of proteins for which there is not an available antibody for western blots.

­
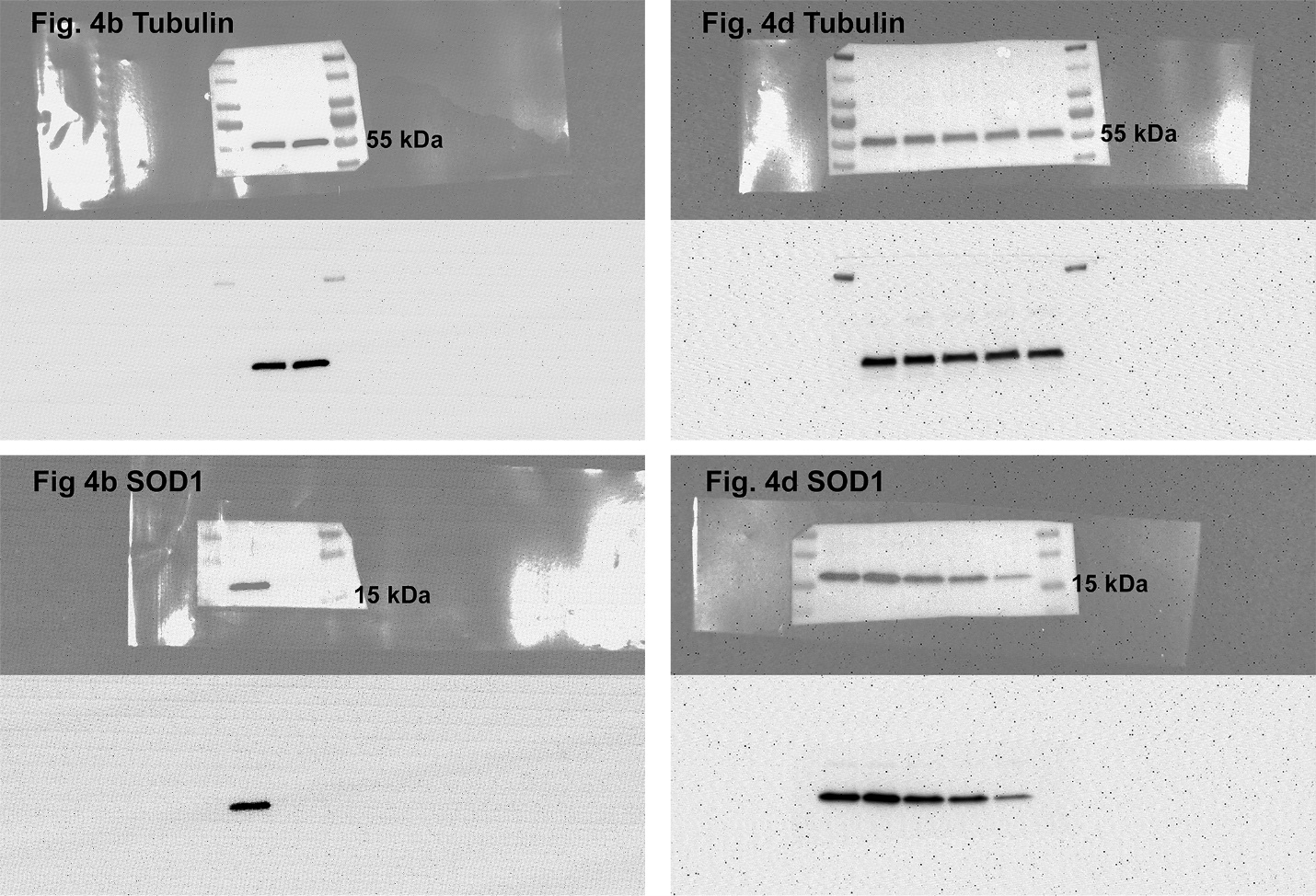


**Supplementary Fig 3) Uncropped Western Blots**

The following images are presented as uncropped references for Fig. 4b and Fig. 4d. Each blot is shown first with a 50% opacity brightfield image overlaid, and on immunostaining alone below. Complete methods may be found in the main text.

**Supplementary Table 1) Physical properties of hairpin sequences tested.**

Based on our preliminary findings (Fig. S1) we estimated that hairpin sequences spanning -27 to -40 kcal/mol would yield attenuators ranging from roughly 50% to 10% original promoter strength. A partial NotI sequence GCGGCCG(C) is integral to the cloning scheme. This serves both as a highly stable base for all hairpin variants as well as the first five nucleotides of the Kozak consensus sequence GCCGC(CACCA). From 5729 unique structures, generated as described in the main methods, we manually selected the following 24. Overall G/C content and positioning of single A/T pair relative to the hairpin loop are kept as uniform as possible, and increments were kept as close to 0.5 kcal/mol as possible (0.54 average). The spreadsheet tables used, along with complete sequences created may be accessed here:

https://bit.ly/millette_frontiers_2022_hairpin_generation

| **HP #** | **Hairpin Sequence** | **Initial ΔG**  **(-kcal/mol)** | **ΔG Step** | **length** |
| --- | --- | --- | --- | --- |
| 24 | CGGCCGCGGGTGGCGGtatactCCGCCACCCGCGGCCG | -40 |  | 38 |
| 23 | CGGCCGCGGGACGGCGtatactCGCCGTCCCGCGGCCG | -39.4 | 0.6 | 38 |
| 22 | CGGCCGCCCAGCCCCtatactGGGGCTGGGCGGCCG | -38.9 | 0.5 | 36 |
| 21 | CGGCCGCCGGAGGGCtatactGCCCTCCGGCGGCCG | -38.3 | 0.6 | 36 |
| 20 | CGGCCGCCCTCGGGGtatactCCCCGAGGGCGGCCG | -37.7 | 0.6 | 36 |
| 19 | CGGCCGCGGCACGGCtatactGCCGTGCCGCGGCCG | -37.3 | 0.4 | 36 |
| 18 | CGGCCGCCGGTGCGGtatactCCGCACCGGCGGCCG | -36.7 | 0.6 | 36 |
| 17 | CGGCCGCGCGAGCGGtatactCCGCTCGCGCGGCCG | -36.1 | 0.6 | 36 |
| 16 | CGGCCGCCCTGGGCtatactGCCCAGGGCGGCCG | -35.6 | 0.5 | 34 |
| 15 | CGGCCGCGGAGCCCtatactGGGCTCCGCGGCCG | -35 | 0.6 | 34 |
| 14 | CGGCCGCCGAGGGGtatactCCCCTCGGCGGCCG | -34.4 | 0.6 | 34 |
| 13 | CGGCCGCGCACGCCtatactGGCGTGCGCGGCCG | -34 | 0.4 | 34 |
| 12 | CGGCCGCGGTGCGGtatactCCGCACCGCGGCCG | -33.4 | 0.6 | 34 |
| 11 | CGGCCGCGTCGGCGtatactCGCCGACGCGGCCG | -32.8 | 0.6 | 34 |
| 10 | CGGCCGCCAGGGCtatactGCCCTGGCGGCCG | -32.3 | 0.5 | 32 |
| 9 | CGGCCGCCTCGGCtatactGCCGAGGCGGCCG | -31.7 | 0.6 | 32 |
| 8 | CGGCCGCCTCGGGtatactCCCGAGGCGGCCG | -31.1 | 0.6 | 32 |
| 7 | CGGCCGCGCACGCtatactGCGTGCGCGGCCG | -30.7 | 0.4 | 32 |
| 6 | CGGCCGCGTGCGGtatactCCGCACGCGGCCG | -30.1 | 0.6 | 32 |
| 5 | CGGCCGCGCGACGtatactCGTCGCGCGGCCG | -29.5 | 0.6 | 32 |
| 4 | CGGCCGCAGCCCtatactGGGCTGCGGCCG | -29 | 0.5 | 30 |
| 3 | CGGCCGCTCGCCtatactGGCGAGCGGCCG | -28.4 | 0.6 | 30 |
| 2 | CGGCCGCTCGGGtatactCCCGAGCGGCCG | -27.8 | 0.6 | 30 |
| 1 | CGGCCGCGTCCGtatactCGGACGCGGCCG | -27 | 0.8 | 30 |

**Supplementary Table 2) Additional reference sequences and their sources.**

| Bap1 Target | GACGAGTTTATCTGTACCTTCA |
| --- | --- |
| Pbrm1 Target | ACCAGAGTCTTTGATCTACAAA |
| Axin1 Target | GGAGCTACAGATACTACTTTAA |
| SOD1 Target 1 | TAGCTGTAGAAATGTATCCTGA |
| SOD1 Target 2 | TGTGGCCGATGTGTCTATTGAA |
| Combined positive control bait | GACGAGTTTATCTGTACCTTCAACCAGAGTCTTTGATCTACAAAGGAGCTACAGATACTACTTTAA |
| Bap1 shRNA | TGAAGGTACAGATAAACTCGTC |
| Pbrm1 shRNA | TTTGTAGATCAAAGACTCTGGT |
| Axin1 shRNA | TTAAAGTAGTATCTGTAGCTCC |
| Luciferase shRNA | TAATATTCCAAAATGATATGAC |
| SOD1 shRNA 1 | TCAGGATACATTTCTACAGCTA |
| SOD1 shRNA 2 | TTCAATAGACACATCGGCCACA |
|  |  |
| Sources of DNA |  |
| Tet-ON and human PGK promoters, and reverse tetracycline transactivator were sourced from Addgene plasmid #41393 |  |
| EMCV IRES was sourced from Addgene Plasmid #27296 |  |
| miRE cassette was sourced from Addgene plasmid #73576 (Vo et al., 2016) |  |
| The EF1a promoter was sourced from Addgene plasmid #11154 (Matsuda and Cepko, 2004) |  |
| mScarlet-I (Bindels et al., 2017) was ordered from IDT as a codon optimized geneblock |  |
